# Personal autonomy development and family functioning of Russian and Azerbaijan adolescents

**DOI:** 10.1192/j.eurpsy.2022.1101

**Published:** 2022-09-01

**Authors:** E. Zakirova, N. Poskrebysheva, A. Babkina

**Affiliations:** Moscow Lomonosov State University, Faculty Of Psychology, Moscow, Russian Federation

**Keywords:** adolescence, personal autonomy, cross-cultural study

## Abstract

**Introduction:**

The development of adolescent’s autonomy is influenced by both: family and culture. Cross-cultural studies show different autonomy development trajectories and culture-specific family organization tendencies. The comparison of autonomy development in different cultures can help in clarifying universal and culture-dependent aspects of autonomy development.

**Objectives:**

The present research studies adolescent’s autonomy in context of family functioning in Azerbaijani (Baku) and Russian (Moscow) adolescents.

**Methods:**

Family Environmental Scale (FES), Method of unfinished sentences to study adolescent’s autonomy fields («I feel independent when…»), The Separation-Individuation Test of Adolescence (SITA) were used in the study with 201 adolescents, aged from 13 to 18.

**Results:**

Family functioning of adolescents from Moscow is less achievement oriented (U-test, p=0,000), family life is less organized (p=0,000) and controlling (p=0,000). Adolescents in Baku consider the value of independence in families higher (p=0,01).

Context analysis of unfinished sentences shows universal categories of autonomy representation (autonomy in specific activities, autonomy as possibility to be alone) and culture specific representations: adolescents from Moscow describe autonomy more like «independence from others», whereas adolescents from Baku describe autonomy as «the presence of others nearby».

Engulfment Anxiety shows negative correlations (р&lt;0,01) with family cohesion (r=0, -0,474), conflict (r=-0,466) and independence (r=-0,326) for all adolescents, with expressiveness (r=-0,490) and achievement orientation(r=-0,286) by Moscow adolescence and with intellectual-cultural (r=-0,249) and recreational family orientation (r=-0,278) by Baku adolescents.

**Conclusions:**

Autonomy development in families has universal aspects (positive effects of cohesion, etc.), but Moscow adolescents are less focused on others and family in their autonomy development.

**Disclosure:**

No significant relationships.

